# Physical morbidity and mortality in male adolescent anorexia: a scoping review

**DOI:** 10.1192/bjo.2021.158

**Published:** 2021-06-18

**Authors:** Craig McEwan

**Affiliations:** Sussex Partnership NHS Foundation Trust (SPFT)

## Abstract

**Aims:**

Anorexia Nervosa (AN) mimics a state of starvation as a result of extreme calorific restriction, often with associated extreme exercise or purging behaviours. The physiological demands are known to lead to a number of health complications and contribute to a significantly increased mortality compared to the general population. Although males account for 10% of the AN population, they are often underrepresented in research. There is a particular gap in evidence for males under 18 despite the unique physiological requirements of adolescence including growth, puberty and achieving peak bone mass.

This review aims to bring together current research on physical health complications in male adolescent anorexia and help understand the knowledge gaps which exist.

**Method:**

A scoping literature review was undertaken between January and March 2020. A single researcher searched OvidSP, psychinfo, relevant grey literature and undertook hand searches of key reference lists. Following PRISMA-SCR protocol, abstracts and articles were screened against inclusion/exclusion criteria to identify relevant papers. Papers were then subjected to critical appraisal and findings summarised using a narrative approach. Key data for blood pressure, pulse and body temperature were pooled and analysed in the context of wider findings.

**Result:**

Data from 219 patients were included from 20 studies. 13 of these studies were case studies or case series, 5 were cross sectional and 2 were cohort studies. Cardiovascular compromise including bradycardia (61%) and hypotension (30.3%) were common and a single episode of cardiac arrest was documented in the literature. Bone density was reduced (Z score ≤1) in 36.7% of cases. A wide variety of single episodes of physical morbidity were also documented including pneumothorax, liver dysfunction, growth retardation and thyroid dysfunction.

**Conclusion:**

This scoping review highlights the physiological compromise experienced by some male adolescents with AN. Guidelines for the identification, assessment and management of physical health complications - including MARSIPAN by the Royal College of Psychiatrists - continues to use data heavily drawn from female-biased populations. Given the evidence summarised, there is concern that in the absence of specific guidance, adolescent males may be at high risk of negative outcomes including acute cardiovascular compromise, osteoporosis and reduced linear growth.

